# Ecological risk evaluation in bottom-surface sediments and sub-surface water in the subtropical Meghna estuarine system

**DOI:** 10.1016/j.heliyon.2021.e08324

**Published:** 2021-11-09

**Authors:** Solaiman Bin Habib, M. Belal Hossain, Md. Solaiman Hossain, Y.N. Jolly, Subrata Sarker

**Affiliations:** a Department of Fisheries and Marine Science, Noakhali Science and Technology University, Noakhali 3800, Bangladesh; b School of Engineering and Built Environment, Griffith University, Nathan Campus, QLD, Australia; c Department of Oceanography, Shahjalal University of Science and Technology, Sylhet 3114, Bangladesh; d Chemistry Division, Atomic Energy Center, Bangladesh Atomic Energy Commission, Dhaka 1000, Bangladesh

**Keywords:** Trace metal, Contamination factor, Spawning ground, River Shad, Risk index

## Abstract

Assessment of elemental contamination is emerging research in the present world. Metals are hazardous to the environment and people's health when metals concentration might exceed the tolerable level. In this research, 12 elements (i.e., Mn, Ni, Cu, Zn, As, Se, Sr, Co, Pb, Fe, Rb, and Ti) were assessed using the energy dispersive X-ray fluorescence (EDXRF) method in water and sediment samples in four (4) different spawning grounds of *Tenualosha ilisha* at the confluence of the Meghna River in Bangladesh. A comparative analysis was performed for the first time among four sampling spots, i.e., Chandpur, Bhola, Sandwip, and Hatiya, assessed all possible risk indices-it is a unique thing. Several risk indices were solved to determine the degree of sediment pollution for all 12 elements, e.g., degree of contamination (C_d_):6.5–7.01, the modified degree of contamination (^m^C_d_): approximately 0.7, the pollution load index (PLI): 0.45–0.51, etc. where all the indices' results showed low or baseline levels of pollution. According to the enrichment factor (EF) computation, slight enrichment of examined metals except Pb and Zn was found. In addition to these, the ecological risk factor (E_r_) found in the following order (pollution level: low): Cu > Pb > Zn among the four stations. Moreover, a spatial incline of metal accumulation was observed among the four spots: Bhola < Sandwip < Hatiya < Chandpur based on the risk index (RI) set value. However, a strong positive correlation (p < 0.05) between Ca and Fe, Ti and Fe, Ti and Mn, Mn and Fe, Fe and Sr were observed while Ca, and Co were strongly negatively correlated (r = minus (-)0.60, p < 0.05). The cluster analysis was performed and got an asymmetrical cluster among the sampling stations. This study recommends assessing the heavy metal concentration in biological samples, particularly in Hilsha fish.

## 1 Introduction

Trace metals are inorganic metals known to be micronutrients. These nutrients are useful and necessary in a minute's concentration for a person (Smith and Garg, 2017). But if their level of presence in the human body is greater than the optimum level, it would be toxic. Zinc (Zn), copper (Cu), selenium (Se), chromium (Cr), cobalt (Co), iodine (I), manganese (Mn), and molybdenum (Mo) are the primary and vital trace elements that humans need in a small amount (Wada, 2004). For example, iron is an essential micronutrient for the human body by transporting oxygen in the blood. Apart from these, elements may play a vital role as enzymatic activity, i.e., various metabolism, hemopoiesis, etc. Considering the importance of calcium, it helps to improve bone and joint health. In terms of potassium, it is crucial for the body's ionic balance.

Moreover, these trace metals are significantly sensitive for their behaviour due to extremely low or incredibly high levels that might affect humans. Notwithstanding this, when the concentration level is beyond the required level, it could negatively affect, e.g. goitre, gout, liver/kidney dysfunction, growth retardation, hypothyroidism, thyroid, CNS disorder, etc. (Wada, 2004). Trace metals can stay at 0.02% of the total body weight, while major elements can account for 96% (Wada, 2004). Some are considered heavy metals with a density of 3–7 gm/cm^3^, but most researchers support this value-specific gravity equal to or greater than 5 gm/cm^3^ (Duffus, 2002).

Some elements can be considered carcinogenic (e.g., Hg, Pb, Cd, Cr, As, etc.), and some are accounted as non-carcinogenic (e.g., Cu, Se, Mn, etc.). Besides, these elements are released into the environment by natural (e.g., rock, weathering, river erosion, volcanic eruptions, etc.) and human-made causes (e.g., industrial, agricultural, medicinal, domestic and atmospheric sources, mining, foundries and smelters, and other metal-based industrial operations) (Christophoridis et al., 2009; Jordao et al., 2002; Lim et al., 2016). Although heavy metal contamination happens naturally, a certain amount of metal remains on the earth's surface. Besides, metal contamination can be increased a considerable amount due to anthropogenic practices as well. Metals pose a risk to the atmosphere because of their characteristics as poisonous, non-biodegradable, and long-lasting. Additionally, it can be dangerous to our CNS and death in extreme cases. Not even the metals are lost after the cooking. Heavy metal causes degradation of the natural quality, deterioration of the ecosystem's health, and threatens near or near the average metal concentration level. These are therefore spread via the transportation phase throughout the world. Metals are presumably first transported to water, then adsorbed, and finally deposited in the sediment (Hakanson, 1980).

Sediments, including metals and metalloids, have often been used as a concierge of chemical pollution. It is the primary sinker of all metals and or depositors (Joksimovic et al., 2011; Luoma and Bryan, 1981). For this reason, it is highly appreciated to examine the contamination status of the sediments of the Meghna river. But metal can come through different processes to the watery environment, such as upwelling, bioturbation, etc. However, a water sample was considered to collect to analyse trace and heavy metals in this research. Metals have been incorporated and transferred from water and sediment into the biological samples including fish, molluscs, crustaceans, and seaweed samples through bioaccumulation (i.e., it is the progressive accumulation of contaminants in an organism, such as heavy metals) and biomagnifications (i.e., it is a mechanism by which a contaminant (such as a heavy metal) raises its concentration as it passes across the food chain mostly in tissues) process.

Furthermore, ecological risk could be increased, and human health risk might be accelerated by ingesting contaminated species. (Ali and Khan, 2019). But this research could be extensive and impressive if biological samples were also accounted for determining the metal transfer ratio from abiotic to biotic environment; this was outside the scope of the study.

However, for understanding the state of aquatic health, water quality parameters were recorded. For example, pH indicates whether the water is acidic or alkaline. Aquaculturists suggest slightly base or neutral pH for aquaculture (6.5–8.5) (Towers, 2015). When the pH value falls from the neutral condition, ocean acidification occurs, and even coral bleaching has destroyed the calcified organisms' body cover. Dissolved oxygen (DO) is an essential quality for the living of aquatic species. The basic primary standard for DO for fish growth is five (5) ppm, except for catfishes. Catfish can survive with their accessory respiratory organs even at two (2) ppm DO, and even some lungfish can suck the oxygen out of the air (Towers, 2015). The recommended DO range is 5 ppm for tropical freshwater and marine fish (Mallya, 2007). Temperature is also an essential factor for fish production. It also influences the metabolism of the fish species. The tropical fish species average ambient temperature varied from 24-32 °C (Towers, 2015). Salinity is the aspect where the effect of seawater in the environment is known. The saltiness of the brackish habitat varied between 5 and 20 ppt. But in some situations, the set of values might be changing from one area to another. The sea region's total salinity is 34.7 ppt, while the freshwater ecosystem is 0 ppt (Ridgway, 1969). Water alkalinity and hardness are considerations for determining water quality, with a typical value of 40–70 mg of calcium carbonate per litre (Towers, 2015). All the criteria of water quality usually have the standard level within the estuarine environment.

The subject of estuarine pollution is of great concern to scientists, local stakeholders, and authorities. Contamination of sediments by heavy metals is a global necessity with a significant proportion in developing countries. The estuarine environment is often regarded as the productive zone in which air-sea gas exchange, nutrient mixing, and water turbulence will continue to be active (Kirk Cochran, 2014). The lower part of the Meghna River estuary is a popular area for *Tenualosa ilisha* (Hilsha, Bangladesh's national fish) breeding ground (Hossain et al., 2020a). Nearly three-fourths of the river and tributaries are related to that system of waterways. Thus, all contaminants may mix with this channel, estuary, and eventually discharge into the Bay of Bengal. Water ecological parameters for Hilsha fish are temperature (21–30 °C), pH (6–7.5), salinity (0–30), and DO (4–6 ppm) (Hossain et al., 2019). Owing to this, it is a burning issue to determine the pollution status of the Meghna estuary. The national fish named Hilsha (*Tenualosa ilisha*) fish migration route is also in this river system. Hilsha is an anadromous fish, and it is migrated from sea area to freshwater for spawning. After hatching and passing childhood, they may again move to the marine area for further development from juvenile to adult. Consequently, Meghna estuarine system is the focal point of pollution assessment for protecting our Hilsha fish spawning and growth development. Hilsha production may deteriorate in some years in Bangladesh, where trace metal pollution might be the one reason.

Further research is suggested to determine whether heavy metal pollution has any effect on Hilsha production in Bangladesh. This research collected the water and sediment samples (ecological indicators) from the four different spots in the Meghna estuary. Our main intention was to determine the condition of ecosystem health. However, the specific objectives of the research are given below-

• To find out the estuarine water quality parameters
• To evaluate the level of heavy metal effluence in sediment and surface water of Meghna estuary.
• To assess the sediment pollution indices
• To determine the ecological risk factor and index using the established equations
• To analyze the recorded data with international standards (e.g., sediment quality guidelines (SQG), USEPA, etc.) and other authors

## 2 Materials and methods

### 2.1 Site selection

**Figure 1:**
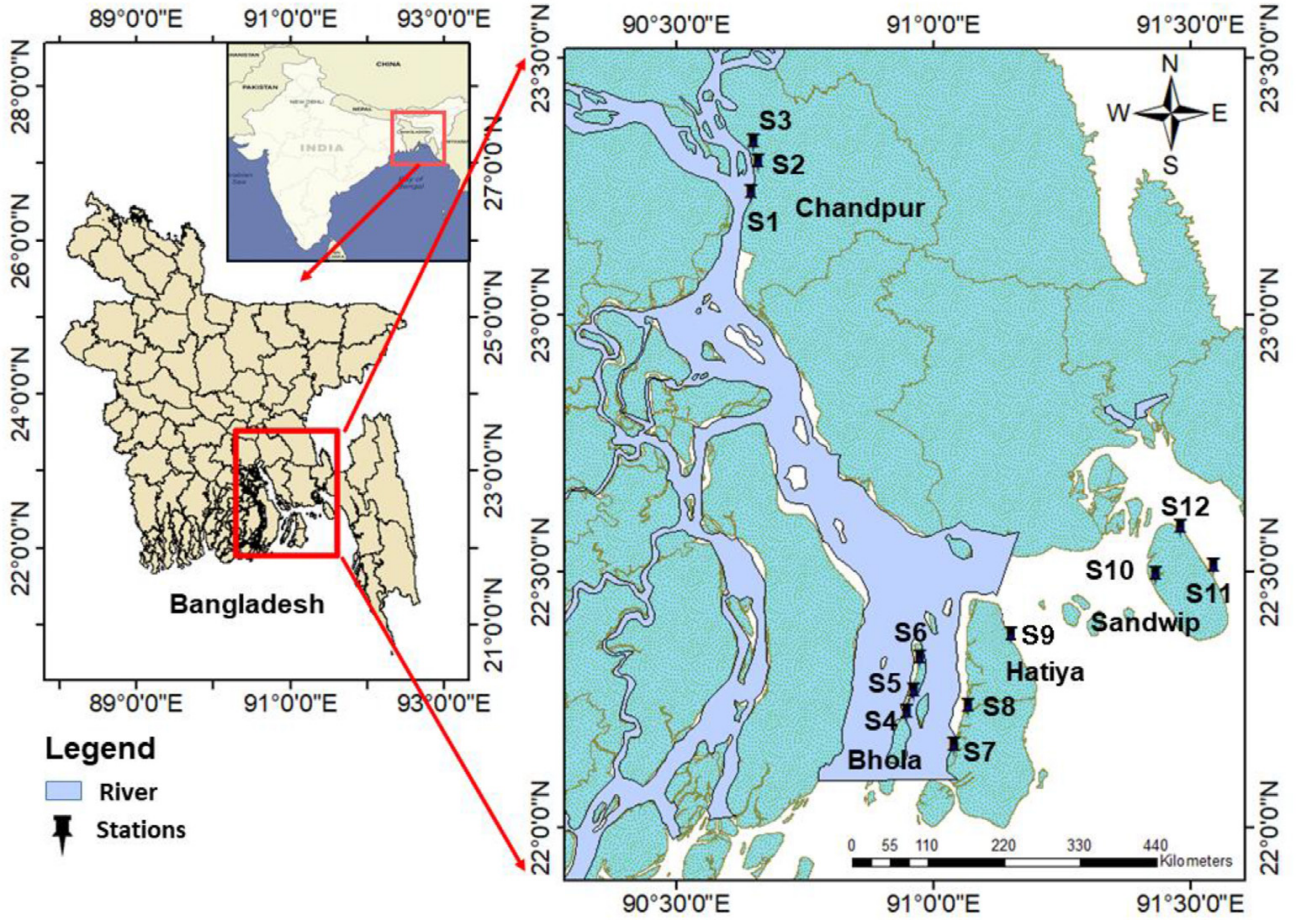
Study area map emphasizing 12 stations in Meghna river channel (Hossain et al., 2020a).

The study was conducted on the lower part of Meghna Estuary, connected to the northern Bay of Bengal. The GBM (Ganga, Brahmaputra, and Meghna) river system is the most extensive. Among the three rivers, the Meghna river is in the lower part and connected to the Bay of Bengal. Almost all the rivers and tributaries' of Bangladesh and even Indian rivers are connected to the Meghna river (Li et al., 2011). Besides, all the sediment and wastage falling have a final destination: rivers, including the Meghna river, ultimately to the Bay of Bengal. So, there is a chance to pollute the environment. Apart from these, the selected site has significant value for considering the spawning ground and habitat of the national fish of Bangladesh named Hilsha (*Tenualosa ilisha*). Considering the Hilsha migration path emphasizing spawning, four important spots and three sampling points from each site were ultimately chosen (12 sampling points in total) in the Meghna river named Chandpur, Bhola, Hatiya, and Sandwip. Besides, 1.5–2 km distance was maintained among the sampling points. The detail of the sampling area map is described in Figure 1.

### 2.2 Water quality parameters' data

Water multi-parameters were measured by hand-held digital meters (HANNA Test kits, Hanna instruments Ltd., Germany), and it was done in the sampling area instantly. First of all, a water sample was collected from the 12 stations by a plastic water bucket, and then 500 mL was poured into a beaker. After that, the probe of the digital meter was immersed and waited 5 min for stability, and finally, data were recorded. Similarly, twelve (12) water quality parameters including pH, temperature, DO, and alkalinity detected from the 12 sampling points where all the determined parameters were considered water health indicators.

### 2.3 Sample collection, preparation, analysis and quality control measures

Water samples were collected from 12 sampling points during the monsoon season in the year of 2017. A boat was used to reach our sampling points and used a plastic bottle for collecting the sub-surface sample water (below 5 cm from the surface for avoiding surfactants). Before this, sampling bottles were washed with de-ionized distilled water and finally rinsed with sample water. Furthermore, duplicate samples were collected from each point to avoid any potential error during the sampling process. After sample collection, 5% of HNO_3_ (pH = 2) was added as an inhibitor to prevent the water's heavy metals decay. After proper labelling of the sample bottle, the next step was to transport these to the laboratory with an icebox and stored them at 4 °C for trace metal detection. Ekman grab sampler was used for collecting sediment samples from the selected locations. Duplicate samples were also collected to avoid the error of the sampling process. However, samples were stored in plastic bags and brought to the laboratory for further analysis. Here we have incorporated the sample analysis in brief. Because the raw data of this research has already been published in a "Data in Brief" journal where the methodology was explained in details, now this data article can be used as a supplementary file with this manuscript (Hossain et al., 2020a).

However, before analyzing the water sample, sampled water was filtered by 0.45 μm filter paper (Sigma-Aldrich, USA). In the next step, 1 g C_6_H_10_O_5_ (cellulose) was added and dried by a water bath (98 °C). Evaporated samples were further dried using an IR lamp (70 °C) for two hours until constant weight. The dry mass was then transferred to a carbide mortar for homogeneous mixing and ground to a powder. The fine powder was used to prepare a pellet by a pellet maker (Specac, UK). Finally, the pellet was kept on the XRF system and the element's concentration was measured as spectrum analysis. In terms of sediment samples, first of all, samples were kept in porcelain dishes individually. After that, the sample was dried until no moisture was present using an oven (70 °C). Finally, a pellet was prepared and kept on an XRF system for metal detection like water sample analysis. For QA/QC, the standard reference material, i.e., Marine sediment, IAEA 433, was used and found precision level was within 94–106%.

### 2.4 Evaluation of sediment contamination

#### 2.4.1 Basic contamination index

To identify the contamination state of sediments, the contamination factor (CF) and the degree of contamination (CD) are employed. The CF and CD are estimated using Hakanson's postulated Eqs. (1) and (2) for Cf and Cd, accordingly (Hakanson, 1980).

(1) $$C_{f}^{i}=\frac{Measured\;concentration,\;Co}{Background\;concentration,\;Cn}$$

(2) $$C_{d}=\overset{n}{\underset{i=1}{\sum }}\;C_{f}^{i}$$

Where the element's baseline value is equal to the average value of the world's surface rock. Cu, Zn, Pb, Ti, Fe, K, Ca, Sr, Zr, and Rb, respectively, have average background concentrations of 45, 95, 20, 4600, 47200, 26600, 22100, 300, 160, and 140 in shale (Turekian and Wedepohl, 1961). The contamination factors were divided into four groups, with different values indicating varying levels of contamination, such as C_f_ < 1 indicating low contamination and 32 > Cd indicating extremely high contamination, however the precise categories were discussed elsewhere (Hakanson, 1980; Hossain et al., 2021a, 2021b).

#### 2.4.2 Advanced tools and/or indices for the determination of the sediment contamination

The formula no. 3 by Abrahim and Parker is being used to define $mC_{d}$ (Abrahim and Parker, 2008).

(3) $$mC_{d}=\frac{\sum_{i=1}^{n}C_{f}^{i}}{n}$$

$mC_{d}$ is also classified into various categories where *mC*_*d*_ < 1.5 signifies nil to very low and *mC*_*d*_ ≥ 32 indicate ultra-high degree of contamination respectively, however, the detailed category was described elsewhere (Abrahim and Parker, 2008; Hossain et al., 2021a, 2021b).

According to Tomlinson (Tomlinson et al., 1980), Pollution Load Index (PLI) is defined as the nth root of the multiplications of the concentrations of the metals. Based on the calculative value of the PLI index following Eq. (4), the state of the sediment contamination can be comprehendible whether baseline or deteriorated (Hossain et al., 2021a, 2021b).

(4) PLI = (CF_1_ × CF_2_ × CF_3_ × … CF_n_)^1/n^

Where CF is the contamination factor which is calculated by the following Eq. (1).

The Geoaccumulation Index (I_geo_) was used to calculate metals content in examined sediments by comparing them to undisturbed or crustal sediment (control) levels. After calculating Eq. (5), numerous Igeo values indicate different pollution status, for example, I_geo_ < 0 signifies unpolluted and I_geo_ > 5 indicates very strongly polluted (Muller, 1969). In total I_geo_ values were classified into seven categories which is described in elsewhere (Hossain et al., 2021a, 2021b).

(5) $$\;I_{geo}=log_{2}\left(\frac{C_{n}}{1.5B_{n}}\right)$$

Where C_n_ is the measured concentration of the sediment for metal (n), B_n_ is the geochemical background value of metal (n), and factor 1.5 is the possible variations of background data due to lithogenic impacts (Rabee et al., 2011).

Because iron (Fe) is the fourth central element in the earth's crust and is rarely contaminated, it was utilized to calculate enrichment factor (EF) in this study. Because along with its noble qualities, Fe is the best-normalized metal. Furthermore, the geochemistry of Fe is nearly identical to that of all toxic elements, and it is equally dispersed throughout the sediments, among other things (Abolfazl and Ahmad, 2011). The number of experts were competent in normalizing metal pollution in river and coastal sediments by using Fe (Neto et al., 2000; Zhang et al., 2007). The EF is calculated by following Eq. (6) (Zoller et al., 1974).

(6) $$Enrichment\;Factor\;\left(EF\right)=\frac{M_{x}/F{e_{x}}_{sample}}{{\left[M_{ref}/Fe_{ref}\right]}_{Background}}$$

The elemental concentration in the sample is denoted by the letter M_x_. The Fe concentration in the sample is denoted by the letter Fe_x_. M_ref_ stands for the elements content in the world's average shale, while Fe_ref_ stands for the average Fe shale. Different EF values indicate different pollution status EF < 1 indicates no enrichment, and EF > 50 is extremely severe enrichment (Abolfazl and Ahmad, 2011; Hossain et al., 2021a, 2021b; Qiao et al., 2013).

### 2.5 Ecological risk calculation from metal contamination in sediment

Potential Ecological Risk (PER) is calculated to evaluate the hazard of an ecosystem's environment. Ecological risk factor and risk index were determined by Eqs. (7) and (8) respectively.

(7) $$\;E_{r}^{i}=T_{r}^{i\;}C_{f}^{i}$$

(8) $$R_{I}=\sum E_{r}^{i}$$

Where $\;E_{r}^{i}$ is ecological risk factor, $C_{f}^{i}$ is contamination factor (see Eq. (1)), and $T_{r}^{i\;}$ is the toxic response factor. The toxic response factor values are 5, 1, and 5 for Cu, Zn, and Pb, respectively (Hakanson, 1980). Moreover, the methods above are also developed by Hakanson (1980). However, different $\;E_{r}^{i}$ and RI values represent different risk categories including $\;E_{r}^{i}$ <40 for low, $\;E_{r}^{i}$ ≥320 for very high risk and $R_{I}$ ≥320 for very high risk (Hakanson, 1980; Hossain et al., 2021a, 2021b).

## 3 Results and discussions

In this research, water and sediment samples were assessed to determine the elements, including trace metal, heavy metal, metalloid level of the Meghna estuarine ecosystem. These two samples are significant objects for determining ecosystem health. However, the findings of this study were compared with different international guidelines and the calculation of several pollution indices to assess the current degree of pollution.

### 3.1 Water quality parameters', Meghna Estuary

Essential physicochemical parameters were measured in the water sample of the studied area and found elsewhere (Hossain et al., 2020a). The water pH was recorded in twelve samples average 7.12 ± 0.27, whereas soil was 6.33 ± 0.41; both pH values are within the preferred range (6.5–8.5) for aquaculture. The temperature was found at 27.974 ± 2.937 degrees Celsius; it also matched with a standard value. Salinity was found in almost 0 (0.14 ± 0.10), meaning a freshwater environment. DO was found 5–9.5 mg/L in all samples due to present current flow, air-water gas exchange continually. In contrast, the study area's alkalinity and hardness were 83.75 ± 21.76 and 204.67 ± 98.28, respectively, which were crossed the standard limit.

### 3.2 Trace metals concentration in the water sample

**Figure 2:**
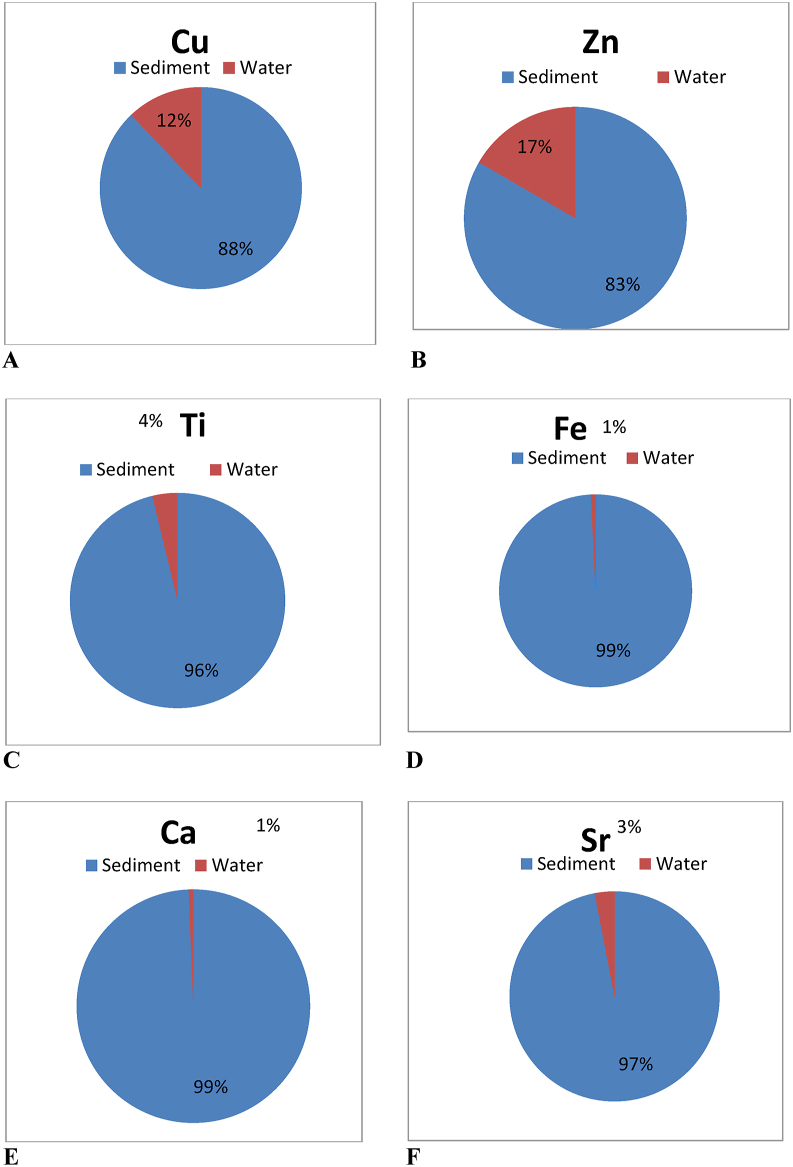
(A to F) Element accumulation percentage between water and sediment sample.

In total eleven elements (Ca, Ti, Mn, Fe, Co, Ni, Cu, Zn, As, Se, and Sr) were determined in the water sample in 2017 during monsoon season (Jun–Aug) where has metal (Ca, Ti, Mn, Fe, Co, Ni, Cu, Zn, and Sr), metalloid (As), and non-metal (Se) and all elements have great significance. For instance, some elements are considered as an essential element in a certain amount for living species like Ca, Fe, and Zn; some are called as toxic elements including As, Co and Ni and some are considered as rare elements including Ti and Sr. However, all the studied elements are under the category of the aquatised cations. No anions or negatively charged elements were considered for this study. In this research, total elemental concentration was determined instead of speciation like total Fe was counted instead of Fe^2+^ or Fe^3+^. The element accumulation percentage was 1–12% in the water sample, whereas 88–99 % was in the sediment sample, as shown in Figure 2. Generally, element concentration was lower in water than sediment because sediment is the final deposition place. But this study found it very low in the water sample due to the monsoon season. During the rainy season, the freshwater influx added to the ecosystem, water might be diluted more, and metals deposited in the bottom sediment (Hossain et al., 2020c).

All the element's concentration in the water sample is described in elsewhere (Hossain et al., 2020a). Bhola's sampling spots were polluted maximum among the four spots in Ca, Ti, Mn, and Fe but *vice-versa* in Chandpur stations. The second most polluted site was Sandwip considering the following metals, i.e., Co, Ni, Cu, and Sr. However, selenium (3.88–4.71 μg/mL) concentration in the water samples was more or less similar for all stations. However, all the elemental concentrations exceeded the international drinking water standard and local guidelines (Table 1). Besides, manganese (Mn) and arsenic (As) were found a high concentration compared to the mentioned literature shown in Table 1. In contrast, the rest of the elements contamination in the studied area was varied with other authors' research (Table 1).

**Table 1:** Comparison of heavy metals in water (μg/L) with different local guidelines and other studies in the world.

Locality/Guideline	Mn	Ni	Cu	Zn	As	Se	Sr	Co	References
Meghna estuary, Bangladesh	17.63	7.51	5.24	6.90	4.39	4.25	5.93	41.32	Present Study, 2017
Meghna Estuary, Bangladesh	NA	10.5	10.2	5.0	2.1	NA	NA	NA	Raknuzzaman et al. (2016)
Chittagong port, Bangladesh	NA	9.0	20.3	5.5	2.3	NA	NA	NA	Raknuzzaman et al. (2016)
Bakkhali Estuary, Bangladesh	NA	5.1	23.9	12.4	5.9	NA	NA	NA	Raknuzzaman et al. (2016)
Danjiangkon Reservior, China	5.69	1.73	13.32	2.02	11.08	NA	NA	1.08	Li et al. (2008)
Izmir Bay, Turkey	NA	6.23–34.63	0.25–88.7	32.7–586	NA	NA	NA	NA	Akinci et al. (2013)
World standard drinking water guidelines	0.4	0.07	2	<3	0.01	0.01	NA	NA	WHO (2011)
Bangladeshi drinking water standards	0.1	0.1	1.0	5.0	0.05	0.01	NA	NA	DPHE (2020)

### 3.3 Trace elements concentration in the sediment sample

Trace element was assessed in a sediment sample from four critical spots in the Meghna estuary and described in elsewhere (Hossain et al., 2020a). The sediment sample's average potassium and calcium concentration was 16750.83 mg/kg and 16984.17, where Bhola stations were mostly polluted than the other three spots. In terms of titanium, Chandpur stations were maximumly contaminated (2950.25 ± 59.79 mg/kg), whereas the minimum was found in the Sandwip station. Hatiya station was more enriched with Rb, Fe, and Sr comparison to others. Besides, no apparent significant data variation among the sampling spots for the following metals, i.e., Pb, Cu, and Zn. Our finding results for especially three metals (Pb, Cu, and Zn) were matched with the metal concentration data in a sediment sample from Krishna River, India (Ramesh et al., 1989). However, the findings of this research is compared with the world average shale (Turekian and Wedepohl, 1961) data and sediment quality guidelines (MacDonald et al., 2000) data, as shown in Table 2. All the studied metal concentration was still below the average shale, but Cu and Rb concentration almost touched the average shale. It is assumed that these two metal concentrations might exceed the world average after a few years. According to sediment quality guidelines (SQG), the concentration of copper (38.1 mg/kg) has already topped over the TEL, threshold effect concentration (31.6 mg/kg). Besides, for more clarification of pollution status of the study site, the several established sediment pollution indices and ecological risk indices were solved (Hakanson, 1980).

**Table 2:** Comparison of heavy and trace metals in sediment (mg/kg) with different local guidelines and other studies in the world.

Locality/Guideline	Pb	Cu	Zn	Fe	Sr	Rb	Ti	References
Meghna estuary, Bangladesh	9.81	38.10	34.64	23961.5	190.42	131.45	2745.37	Present Study, 2017
Meghna river, Bangladesh	22	32	110	46500	106	NA	5200	Datta and Subramanian (1998)
Lower Ganges, Bangladesh	15	23	71	38000	98	NA	3700	Datta and Subramanian (1998)
Krisna River, India	9	35	26	25100	253	NA	3158	Ramesh et al. (1989)
Tigris River, Turkey	62.3–566.6	11.2–5075.6	60.1–2396.6	NA	NA	NA	NA	Varol (2011)
SGQ (TEC)	35.8	31.6	121	NA	NA	NA	NA	MacDonald et al. (2001)
SQG (PEC)	128	149	459	NA	NA	NA	NA	MacDonald et al. (2000)
World average shale	20	45	95	47200	300	140	4600	Turekian and Wedepohl (1961)

Note: SQG: sediment quality guideline, TEC: threshold effect concentration; PEC: probable effect concentration.

### 3.4 Sediment pollution indices

In this study, sediment pollution was assessed based on two categories viz i. for assessing the contamination status for each metal in all stations, ii. for vulnerability assessment by ecological risk factor and risk index calculation.

#### 3.4.1 Degree of contamination assessment for all elements in the sediment sample

Contamination factor (C_f_) for all elements, i.e., Cu, Zn, Pb, Ti, Fe, K, Ca, Zr, Sr, and Rb, in the studied area, was assessed found less than 1 value. It indicated that all the low contamination in the region by studied elements. In contrast, only in rubidium trace metals in the sediment sample of Hatiya station were more significant than 1, moderate meaning contamination. Although the C_f_ values for all metals were below 1, this C_f_ value is close to 1 (range 0.7–0.99) for three metals, i.e., Cu, Sr, and Zn. Consequently, this study might suspect that the value of the three metals above' contamination factor might be become > 1 after few years.

C_d_, ^m^C_d_, and PLI indices were calculated for all metals for in-depth justification of the sediment pollution using trace metals concentration (Table 3). The degree of contamination (Cd) value was ranged from 6.5 to 7.01 indicating low contamination status. Besides, a modified degree of contamination was at the minimum range. However, the PLI value was found around 0.50, which means a baseline level of pollution, i.e. it is not in a perfection stage. Many anthropogenic sources have remained in the experimental place (Abrahim and Parker, 2008).

**Table 3:** Degree of contamination, modified degree of contamination, pollution load index, ecological risk factor, and risk index calculation from sediment sample.

Stations	C_d_	CS/PL	mC_d_	CS/PL	PLI	CS/PL	Ecological risk factor, Er				CS/PL	RI	CS/PL
							Cu	Zn	Pb	Cu			
Chandpur	6.77	Low	0.68	Nil to very low	0.49	baseline levels	4.31	0.35	3.1	4.31	Low	7.77	Low
Bhola	6.65	Low	0.67	Nil to very low	0.46	baseline levels	4.36	0.35	1.91	4.36	Low	6.62	Low
Hatiya	7.01	Low	0.7	Nil to very low	0.51	baseline levels	4.20	0.39	2.41	4.20	Low	7.00	Low
Sandwip	6.5	Low	0.65	Nil to very low	0.45	baseline levels	4.06	0.37	2.38	4.06	Low	6.81	Low

**N.B.** CS for Contamination Status/PL for Pollution Degree.

**Figure 3:**
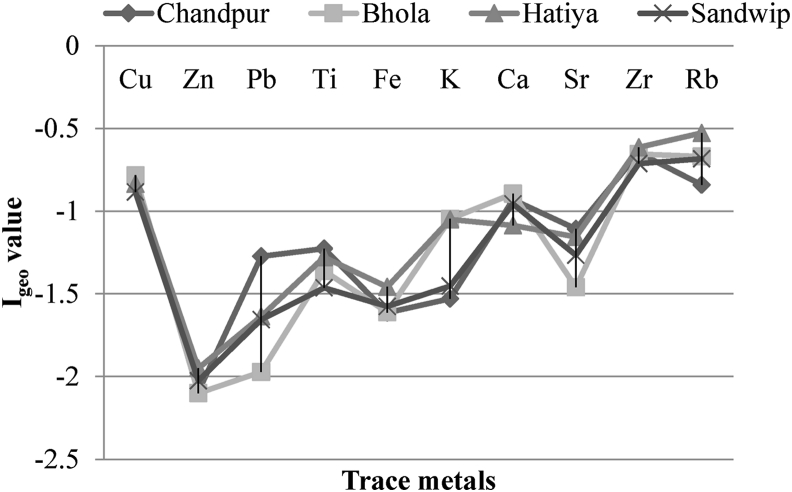
Geoaccumulation index (I_geo_) estimation for four sampling spots.

Apart from these, two essential pollution indices named geo-accumulation index (I_geo_) and enrichment factor (EF) were also calculated. The I_geo_ was found a negative value for all studied metals, which indicates low pollution status, as shown in Figure 3.

In the enrichment factor (EF) estimation, minor enrichment (EF = 1–2) was found for almost all experimented metals in all the stations (Table 4). In contrast, EF values for Zn and Pb for all stations were recorded below 1, meaning no enrichment. However, the lead had only minutely (EF = 1.27) enriched in Chandpur station.

**Table 4:** Enrichment factor assessment for all studied metals in the sediment sample, Bangladesh.

Stations	Trace metal								Pollution level
	Cu	Zn	Pb	Ti	K	Ca	Sr	Zr	
Chandpur	1.76	**0.72**^a^	1.27	1.31	1.06	1.61	1.42	1.95	Minor enrichment
Bhola	1.77	**0.71**^a^	**0.78**^a^	1.19	1.48	1.64	1.11	1.94	Minor enrichment
Hatiya	1.54	**0.71**^a^	**0.88**^a^	1.13	1.33	1.29	1.23	1.79	Minor enrichment
Sandwip	1.62	**0.73**^a^	**0.95**^a^	1.08	1.09	1.54	1.24	1.82	Minor enrichment

a Bold values indicate: No enrichment.

#### 3.4.2 Potential ecological risk estimation

Estimating the ecological risk of heavy metal contamination was proposed as an analytical tool for water pollution control purposes (Rahman et al., 2014). Owing to this, the content of heavy metals in sediments is also increasing. The metals could affect ecological health through this process. Hakanson created a system for evaluating possible environmental risk indices for managing aquatic pollution (Rahman et al., 2014). Besides, the method would also use finding out whether lakes or rivers or oceans are polluted or not and should be given special attention (Hakanson, 1980). This research calculated the ecological risk factor for only three metals among the studied metals due to the toxic response factor's availability (Table 3). The order of potential ecological risk factor of heavy metal in bottom sediments of the Meghna estuary was Cu > Pb > Zn. The potential ecological risk factors E_r_ for Cu, Pb, and Zn were less than 40, which belong to low ecological risk (Hakanson, 1980). Without this, risk index (RI) was also determined and found less than 140 values for all metals, indicating the light pollution area.

### 3.5 Statistical analysis

**Figure 4:**
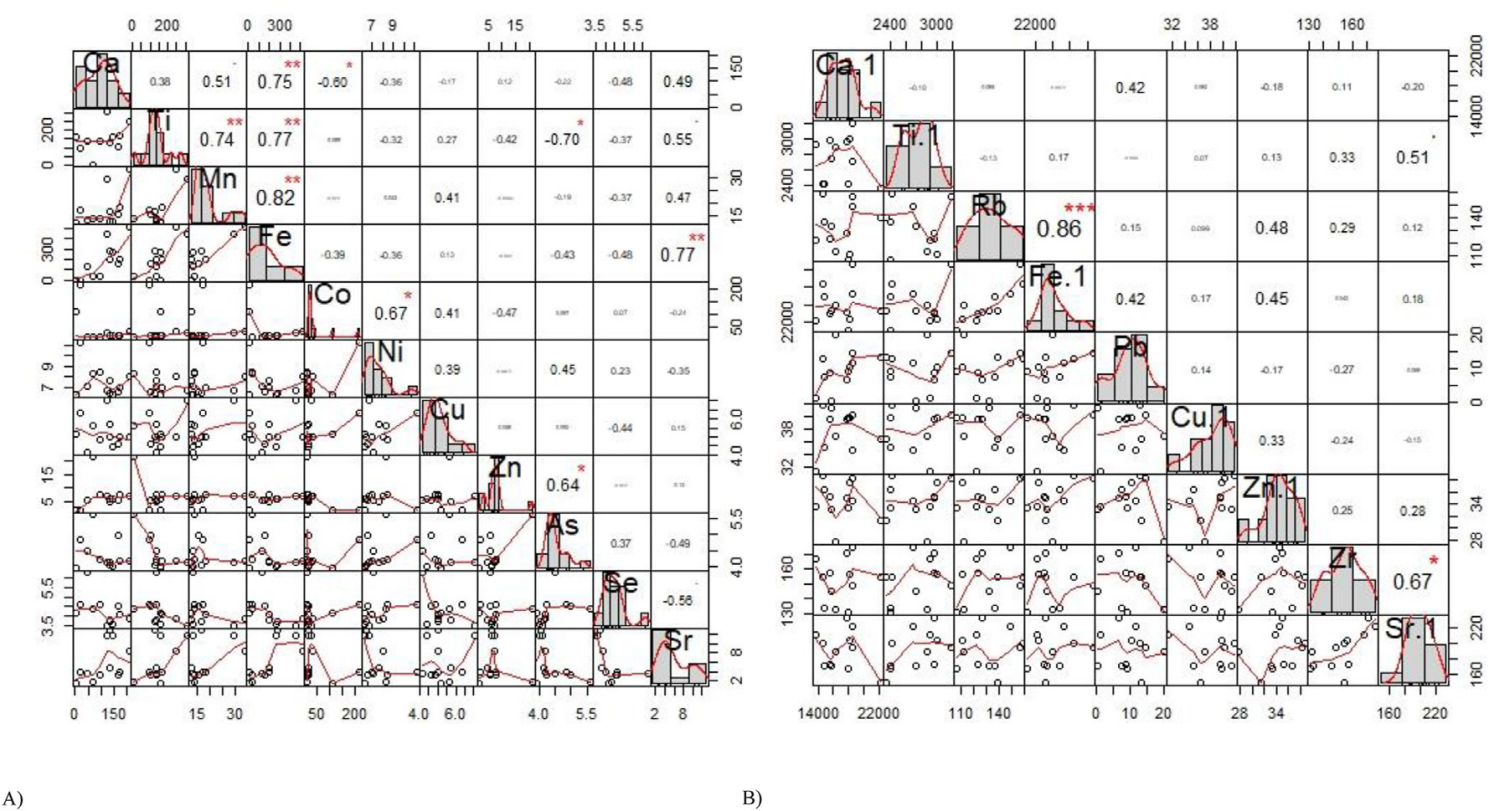
(A and B) Correlation among the trace metals concentration in water (A) and sediment (B).

**Figure 5:**
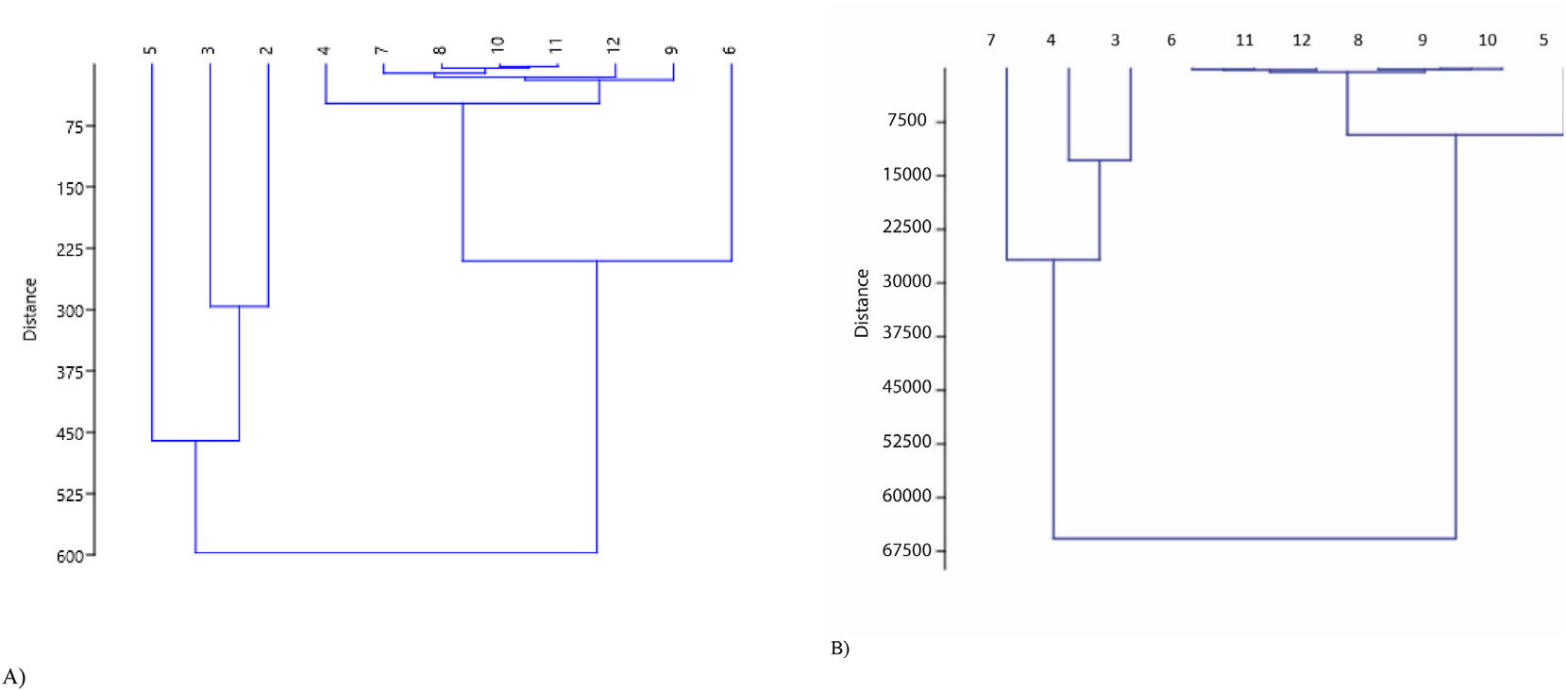
(A and B) Cluster analysis in trace metal concentrations in water (A) and sediment (B).

Multiple correlation analysis (Figure 4) on the elemental data of water found that there is a strong correlation exists between Ca and Fe (r = 0.75, p < 0.001), Ti and Mn (0.74, p < 0.001), Ti and Fe (r = 0.77, p < 0.001), Mn and Fe (r = 0.82, p < 0.001) and Fe and Sr (r = 0.77) = , p < 0.001). The correlation was observed between Ca and Co (r = -0.60, p < 0.05), Ti and As (r = 0.64, p < 0.05), Co and Ni (r = 0.67, p < 0.05), and Zn and As (r = 0.64, r = 0.05). Positive relationship (i.e., positive value of r) indicates increase one metal led to an increase of other metal while vice-versa for negative relationship (i.e., negative value of r). In terms of sediment, slightly different result was found for correlation assessment. For instance, a positive correlation was found between Rb and Fe (r = 0.86, p < 0.001) and Zr and Sr (r = 0.67, p < 0.05). Relation between other metal concentrations is not significantly correlated (Figure 4). Cluster analysis found two major clusters in heavy metal concentrations at different sampling locations (Figure 5). Cluster 1 consists of stations 4, 7, 8, 10, 11, 12, 9, and 6, while the second cluster consists of stations 2, 3, and 5. Two major clusters was found for the heavy metal concentration at different stations in sediments (Figure 5). Cluster 1 consists of station 6, 11, 12, 8, 9, 10 and 5, while cluster 2 consists of station 7, 4 and 3. Interestingly, clusters in-terms of sampling stations found in water and sediments are not symmetrical.

## 4 Conclusion

Trace metals assessment in the water and sediment sample meaning to judge the pollution status in any ecology. In this work, 12 significant elements, including toxic chemicals, in both representatives, i.e., water and sediment from the trendy places in the subtropical area of the Meghna estuary. Elemental concentration was low to medium range among sampling stations. Based on the pollution indices, i.e., C_d_, ^m^C_d_, and PLI, the study site is low or baseline contamination level yet. In terms of enrichment factor index, all the elements show slight enrichment excluding Pb and Zn. However, this study site is moderately polluted for a few metals and low contamination in almost all metals according to international guidelines and the sediment pollution indices' calculated value. The primary pollutants in the study area include agricultural runoff, household waste, industrial effluents, water vehicles waste, and river runoff. According to cluster analysis, all the elements' source is not the same and found asymmetrical cluster. Nevertheless, this study strongly recommends taking a biological sample (fish, molluscs, crustaceans, etc.) for trace metal detection, and Hilsha fish should prioritize future research.

## Declarations

### Author contribution statement

Solaiman Bin Habib: Conceived and designed the experiments; Performed the experiments; Wrote the paper.

M. Belal Hossain: Conceived and designed the experiments.

Md. Solaiman Hossain: Analyzed and interpreted the data; Wrote the paper.

Y. N. Jolly: Performed the experiments; Contributed reagents, materials, analysis tools or data.

Subrata Sarker: Analyzed and interpreted the data.

### Funding statement

This research did not receive any specific grant from funding agencies in the public, commercial, or not-for-profit sectors.

### Data availability statement

Data included in article/supplementary material/referenced in article.

### Declaration of interests statement

The authors declare no conflict of interest.

### Additional information

No additional information is available for this paper.
